# Renal cell carcinoma primary cultures maintain genomic and phenotypic profile of parental tumor tissues

**DOI:** 10.1186/1471-2407-11-244

**Published:** 2011-06-13

**Authors:** Ingrid Cifola, Cristina Bianchi, Eleonora Mangano, Silvia Bombelli, Fabio Frascati, Ester Fasoli, Stefano Ferrero, Vitalba Di Stefano, Maria A Zipeto, Fulvio Magni, Stefano Signorini, Cristina Battaglia, Roberto A Perego

**Affiliations:** 1 Institute for Biomedical Technologies (ITB), National Research Council (CNR), via Fratelli Cervi 93, 20090 Segrate, Milan, Italy; 2 Department of Experimental Medicine, University of Milano-Bicocca, via Cadore 48, 20052 Monza, Milan, Italy; 3 Department of Medicine, Surgery and Dentistry, Pathological Anatomy Unit, University of Milan, Ospedale Maggiore Policlinico, via Sforza 35, 20122 Milan, Italy; 4 Department of Laboratory Medicine, Desio Hospital, University of Milano-Bicocca, via Mazzini 1, 20033 Desio, Milan, Italy; 5 Dipartimento di Scienze e Tecnologie Biomediche, Università degli Studi di Milano, via Fratelli Cervi 93, 20090 Segrate, Milan, Italy

## Abstract

**Background:**

Clear cell renal cell carcinoma (ccRCC) is characterized by recurrent copy number alterations (CNAs) and loss of heterozygosity (LOH), which may have potential diagnostic and prognostic applications. Here, we explored whether ccRCC primary cultures, established from surgical tumor specimens, maintain the DNA profile of parental tumor tissues allowing a more confident CNAs and LOH discrimination with respect to the original tissues.

**Methods:**

We established a collection of 9 phenotypically well-characterized ccRCC primary cell cultures. Using the Affymetrix SNP array technology, we performed the genome-wide copy number (CN) profiling of both cultures and corresponding tumor tissues. Global concordance for each culture/tissue pair was assayed evaluating the correlations between whole-genome CN profiles and SNP allelic calls. CN analysis was performed using the two CNAG v3.0 and Partek software, and comparing results returned by two different algorithms (Hidden Markov Model and Genomic Segmentation).

**Results:**

A very good overlap between the CNAs of each culture and corresponding tissue was observed. The finding, reinforced by high whole-genome CN correlations and SNP call concordances, provided evidence that each culture was derived from its corresponding tissue and maintained the genomic alterations of parental tumor. In addition, primary culture DNA profile remained stable for at least 3 weeks, till to third passage. These cultures showed a greater cell homogeneity and enrichment in tumor component than original tissues, thus enabling a better discrimination of CNAs and LOH. Especially for hemizygous deletions, primary cultures presented more evident CN losses, typically accompanied by LOH; differently, in original tissues the intensity of these deletions was weaken by normal cell contamination and LOH calls were missed.

**Conclusions:**

ccRCC primary cultures are a reliable *in vitro *model, well-reproducing original tumor genetics and phenotype, potentially useful for future functional approaches aimed to study genes or pathways involved in ccRCC etiopathogenesis and to identify novel clinical markers or therapeutic targets. Moreover, SNP array technology proved to be a powerful tool to better define the cell composition and homogeneity of RCC primary cultures.

## Background

The clear cell subtype of renal cell carcinoma (ccRCC) accounts for 85% of all RCCs and occurs as familial or, more often, sporadic forms. It is characterized by recurrent genetic anomalies, like copy number alterations (CNAs) and loss of heterozygosity (LOH), that involve specific chromosomes (chrs) and result in deletions with LOH on chrs 3p (often involving the von Hippel Lindau (*VHL*) locus in 3p26-p25), 6q, 8p, 9p and 14q, and duplications of chrs 5q and 7 [1-3]. Evidences suggest that this peculiar pattern of genomic instability represents a tumor-specific molecular fingerprint useful for diagnostic and prognostic applications [3-5]. However, more work still needs to completely clarify the complex molecular pathogenesis of ccRCC. Although the involvement of the *VHL *tumor suppressor gene has been demonstrated in all familial and in 80-90% sporadic ccRCCs, the remaining 10-20% harbors wild-type *VHL *[6,7], suggesting that, despite their identical histological phenotype, these tumors have an intrinsic molecular heterogeneity that still needs to be unraveled [6,8]. The recognition of this molecular heterogeneity might improve the selection of patients for targeted therapies and allow the identification of specific oncogenes and tumor suppressor genes to be used as novel clinical markers or therapeutic targets.

The current availability of high-throughput platforms to assess molecular changes at genome-wide level might provide an opportunity to achieve this goal. Presently a comprehensive and detailed genomic profiling of DNA alterations is possible by using the single nucleotide polymorphism (SNP) array technology. Unlike CGH technique, the SNP array platform allows the simultaneous analysis of both chromosomal and allelic imbalances [9] and the distinction between LOH associated with CN changes (such as hemizygous deletions) and CN neutral status (often termed as uniparental disomy) [10]. In addition, this technology is able to provide information about the fraction of normal and tumor cells present in the tumor tissue samples [11]. This allows a more complete definition of the complex genetic rearrangements associated with cancer pathologies. Moreover, since the regions of LOH accompanied by deletion (representing the second hit of Knudson's hypothesis) are of particular interest because they may contain genes involved in tumor etiology, the assessment of deletion/LOH areas represents a useful approach to identify regions potentially harboring novel tumor suppressor genes [12]. Accordingly, in a previous study, we used the SNP array technology to characterize the whole-genome DNA profile of a collection of ccRCC tissue samples to find novel chromosomal regions and genes potentially interesting as candidate tumor markers [13].

However, in ccRCC specimens, as in most solid tumor tissues, the molecular analyses may be affected by tissue heterogeneity due to the presence of necrotic areas and non-tumor cells, such as tumor-infiltrating leukocytes, endothelial cells and fibroblasts [14]. With the purpose to increase the quality of data by minimizing this "background noise", several different technical approaches have been explored [15]. Tissue heterogeneity must be considered also when evaluating which are the most appropriate computational tools to process and analyze array-based CN data [11,16].

Thus, to overcome the problem of tissue heterogeneity and to prospectively perform *in vitro *functional studies aimed to better understand ccRCC molecular pathogenesis, it is necessary to have a viable and more homogeneous cell material retaining the phenotypic and genomic profile of original tissue. A possible strategy to face these requirements is to adapt fresh ccRCC tissue specimens to grow *in vitro *as primary cell cultures, which provide a good quality, homogeneous and well-characterized cellular material, enriched with tumor cell component [17] and retaining at the first passages the phenotypic and proteomic profile of the corresponding tissues from which they derive [15,18,19]. Hence, primary cultures represent a better *in vitro *tumor model than stable cell lines, that can be even very different from the original tissues and thus not at all representative [6]. Anyway, the reliability of data obtained from primary cultures strictly depends on their careful cytological characterization, especially regarding possible cell contaminations that might influence data interpretation with misleading effects.

In this study, we investigated whether ccRCC primary cultures, established from surgical tumor specimens and phenotypically well-characterized, maintain the DNA profile of parental tumor tissues and allow a better discrimination of CNAs and LOH with respect to original tissues. Till now, the comparison of DNA profile between tumor primary cultures and parental tissues has been reported, with discordant results, in melanoma [20], neuroblastoma [21] and glioblastoma [22], using either SNP array or array-CGH techniques, and in RCC [23] only using traditional CGH on metaphase spreads and short-term primary cultures not extensively characterized.

## Methods

### ccRCC primary culture preparation and immunophenotypic characterization

Nine ccRCC primary cultures were established from corresponding surgical tissue specimens of ccRCC cases (Table 1). Patients were enrolled in the research project at Policlinico Hospital (University of Milan, Italy) and provided informed consent for the research use of leftover material. Study protocol and procedures were approved by the local ethic committee. All RCC cases were diagnosed as "clear cell" subtype independently by two pathologists with expertise in kidney cancer. Prior to surgery, a whole blood sample was collected for each case and stored at -20°C. Immediately after surgical removal of the kidney, sections of fresh tissue samples enriched in tumor component by needle dissection were both stored at -80°C and collected in cold DMEM medium supplemented with 10% fetal calf serum (FCS), 1% penicillin/streptomycin, 1% amphotericin and 1% glutamine (Culture Medium) and kept at 4°C until processing (within 24 hrs). After removal of adipose and necrotic areas, tumor tissues were mechanically minced in 1 mm^3^-fragments and digested with 25 mg/ml collagenase type IV (Sigma-Aldrich, St Louis, MO, USA) in DMEM medium for 2 hrs at 37°C, vigorously vortexing every 15 minutes. Then, samples were washed three times in PBS at 4°C and plated in 60 mm-Petri dishes or on glass cover slips in the Culture Medium and incubated at 37°C in 5% CO_2_. Medium was changed twice weekly and cells were 1:3 splitted when reaching 90% confluence. Cell morphology was observed in contrast phase, at 100× magnification, by Olympus CK40 inverted microscope (Olympus Corporation, Tokyo, Japan).

**Table 1:** Clinical data of the nine clear cell RCC cases from which primary cultures have been prepared.

Case	Gender	Age (years)	Tumor size (cm)	Tumor stage (pT)/Grade
50PC	M	51	5.7	pT1/G2
59RG	M	72	5	pT1/G2
60CC	M	78	11.5	pT3/G3
61FG	M	56	5	pT1/G2
66SML	F	48	9	pT2/G2
70LS	M	71	4.4	pT1/G2
73PG	F	75	2.5	pT1/G3
80MLa	F	78	6.2	pT3/G2
81BPG	M	69	3.3	pT1/G2

For immunofluorescence microscopy, ccRCC cells grown on glass cover slips were fixed for 30 min in 4% paraformaldehyde PBS buffer, pH 7.2, at 37°C, rinsed with PBS, pre-incubated for 15 min in GDB buffer (0.02 mol/l sodium phosphate buffer, pH 7.4, with 0.45 mol/l NaCl and 0.5% BSA) containing 0.3% Triton X-100, and incubated with mouse monoclonal antibodies (mAbs) against pan-cytokeratin (clone MNF-116, Dako, Glostrup, Denmark; dilution 1:200), vimentin (clone V9, Dako; dilution 1:200), carbonic anhydrase IX (CA9) (clone M75, dilution 1:50; a gift from Prof. Pastorekova, Institute of Virology, Slovak Academy of Sciences, Bratislava, Slovak Republic) and FITC-conjugated mAb against CD13 (clone CBL 169F, Chemicon, Billerica, MA, USA; dilution 1:25) for 2 hrs at room temperature. After washing in PBS, cover slips were incubated for 1 hr with goat anti-mouse Alexa-Fluor488-conjugated IgG secondary antibody (Molecular Probes, Invitrogen Life Technologies, Carlsbad, CA, USA; dilution 1:100). Nuclear counterstaining was performed by incubation for 5 min with 1 μM DAPI (Sigma-Aldrich) in PBS buffer. Immunofluorescence micrographs were obtained using a Zeiss Axiovert 200 inverted microscope (Zeiss Inc., Oberkochen, Germany), at 400× magnification, equipped with a CoolSNAP HQ camera driven by Metamorph software. For flow cytometry analysis, cells at the first confluence (p1) were detached from plates with 0.25% trypsin-EDTA (Sigma-Aldrich), rinsed and incubated for 15 min in PBS with 5% FCS. For CD13 and CA9 staining, cells were incubated for 15 min at room temperature with the specific mouse mAbs. Cytokeratin and vimentin staining was performed using the specific mouse mAbs after permeabilization with IntraStain solution (Dako). Cells were then incubated with goat anti-mouse Alexa-Fluor488-conjugated IgG secondary antibody (Molecular Probes, Invitrogen Life Technologies) for 30 min at 4°C and counted by the FACSCalibur flow cytometer and CellQuest software (BD Biosciences) until 30,000 events were acquired.

### Western Blot analysis

For each case, cells plated in a 6 mm-Petri dish were lysated when at the first confluence (p1), and the extracted proteins quantified by the Bio-Rad microassay (BioRad, Hercules, CA, USA), as previously described [18]. 30 μg proteins were separated on NuPage 4-12% Bis-Tris pre-cast gels (Invitrogen Life Technologies) and blotted onto nitrocellulose membranes probed in 10 mM Tris-HCl, pH 8, and 3% BSA, with mouse mAb against CA9 (clone M75, dilution 1:50). Detection was performed using a secondary antibody coupled with horseradish peroxidase for 1 hr at room temperature and the SuperSignal West Pico detection system (Pierce, Rockford, IL, USA).

### DNA extraction and target preparation for Affymetrix SNP Arrays

The genomic studies were performed on all ccRCC primary cultures at first confluence (p1); in addition, for 80MLa and 81BPG cases also cultures at second confluence (p2) or at second (p2) and third (p3) confluences were characterized. Genomic DNA was extracted from primary cell cultures by QIAmp DNA Mini kit (Qiagen, Hilden, Germany), according to manufacturer's cultured cell protocol. DNA from tumor tissues and autologous whole blood samples, used as normal control, was extracted using a standard proteinase K cell lysis and phenol-chloroform procedure. Samples were quantified by ND-1000 spectrophotometer (NanoDrop Technologies, Wilmington, DE, USA) and checked by electrophoresis on 0.8% agarose gel. Starting from 250 ng, DNA samples were processed using the GeneChip^® ^Human Mapping 50K Hind Assay kit (Affymetrix, Santa Clara, CA, USA), according to manufacturer's protocol, and hybridized onto GeneChip^® ^Human Mapping 50K Hind Arrays. Intensity signals were acquired by Affymetrix GeneChip^® ^Scanner 3000 7G and quantified by GTYPE v4.1 software (Affymetrix), using the BRLMM algorithm to assign SNP calls and to generate CHP files. A SNP call rate greater than 95% was considered as "good quality" threshold.

### Genome-wide copy number and LOH profiling in ccRCC primary cultures and tumor tissues

To assess copy number alterations (CNAs) and loss of heterozygosity (LOH), two different software CNAG (version 3.0) and Partek Genomics Suite (version 6.5) were applied. CNAG is a well known software commonly used to analyze SNP array derived-CN data in tumor samples [24]. The AsCNAR (allele-specific copy-number analysis using anonymous reference) algorithm was applied to perform a "self-reference paired analysis", by comparing each culture and corresponding tumor tissue to the matched blood sample. All resulting profiles (global log ratio CN profiles; allele log ratio CN profiles; Hidden Markov Model (HMM)-inferred CN state; HMM-inferred LOH) were visually inspected to identify regions affected by aberrations. To increase data reliability, we chose to include only CNAs longer than 2 Mb (resolution limit of 50K SNP array platform). CNAG software was also used to obtain a genome-wide map of LOH events occurring in each culture or tumor tissue with respect to matched blood. Only the LOH events defined as statistically significant by CNAG (with LOH likelihood higher than 30, as default threshold, and thus visualized in the HMM-LOH track) and covering regions longer than 2 Mb were considered.

The same paired analysis was performed using Partek Genomics Suite software (Partek Inc., St Louis, MO, USA), starting from CEL intensity files and applying the two alternative algorithms Hidden Markov Model (HMM, the same used by CNAG) and Genomic Segmentation (GS). For HMM analysis, default parameters were adopted; for GS analysis, the default window of 10 contiguous SNPs was maintained, Signal to noise ratio at 0.5 and p-value at 0.001 were also adopted.

To estimate the global concordance between the genomic profile of each primary culture and its parental tissue, a paired whole-genome CN correlation was calculated, starting from the CNAG "HMM-CN state" data (SNP copy number status inferred by HMM), and applying Spearman's regression method. SNP call concordance index and Spearman's correlation between the CNAG SNP allelic calls of two matched samples were also calculated. For SNP call concordance, a contingency table of the counts for each combination of the four genotype categories (AA, AB, BB, NoCall) for culture (in row) and matched tissue (in column) was accomplished. The concordance index was calculated as a ratio between the sum of principal diagonal counts and 56,859 (total number of real informative SNPs on 50K Hind arrays), multiplied by 100. A SNP call concordance higher than 60% for two related samples was adopted as threshold. All statistical analysis were performed in R environment.

## Results

### Phenotypic characterization of ccRCC primary cultures

**Figure 1:**
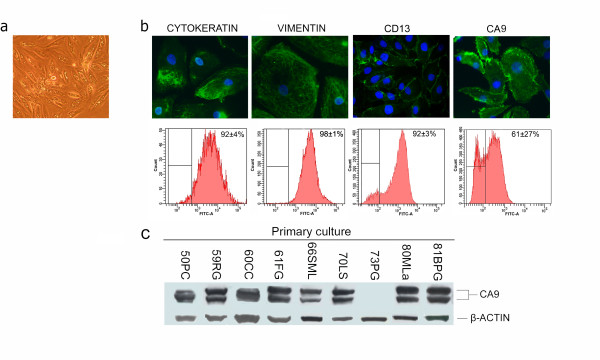
**Phenotypic characterization of ccRCC primary cultures**. (a) Representative cellular morphology during *in vitro *growth. 100× magnification. (b) Representative micrographs of immunofluorescence staining (top) and FACS analysis (bottom) of pan-cytokeratin, vimentin, CD13 and CA9. DAPI counterstains nuclei in blue. 400× magnification. The positivity percentages for the different markers are reported in the FACS analysis as mean value (± SD) of the nine cultures. (c) Western Blot analysis of CA9 in all ccRCC primary cultures. B-actin was used as internal control.

The nine ccRCC primary cultures established from surgical tissue specimens reached the first confluence (p1) in 8.0 ± 2.1 days. They grew well till reaching the fourth confluence, and then began to slow down their growth rate. In particular, 80MLa and 81BPG cultures reached the second confluence (p2) after 13 days, and 81BPG culture reached the third confluence (p3) after 20 days. Cells exhibited heterogeneous epithelioid morphology, were able to form foci and presented cytoplasmic vacuoles frequent in clear cell RCC subtype during *in vitro *growth (Figure 1a). Cytoplasmic staining specific for the epithelial cytokeratin and for vimentin, a mesenchymal marker expressed also in the epithelial RCC cells, both *in vivo *and *in vitro *[18,25], and specific expression of the proximal tubular marker CD13 were observed in more than 90% of cells, as assessed by immunofluorescence and FACS analysis, according to the proximal tubular origin of ccRCC (Figure 1b). Immunofluorescence and FACS analysis were performed also with the monoclonal antibody against the transmembrane carbonic anhydrase IX (CA9) protein, one of the most used biomarkers for ccRCC [26,27]. Our ccRCC primary cultures showed the typical pattern of membrane fluorescence and more than 60% of cells were positive for CA9 (Figure 1b). Moreover, protein lysates analyzed by Western Blot with anti-CA9 antibody showed the expected doublet at about 55 kDa in all samples, except for 73PG culture (Figure 1c). On the whole, these results confirmed the neoplastic phenotype of our tubular primary cultures.

### Genome-wide assessment of CNAs and LOH in ccRCC primary cultures

The Affymetrix 50K SNP Array platform was used to perform the whole-genome SNP profiling of 12 samples from ccRCC primary cultures, nine at the first confluence (p1), two at the second confluence (p2), and one at the third confluence (p3). In addition, 9 samples from the corresponding original tumor tissues and 9 autologous blood samples were analyzed. We obtained an average SNP call rate equal to 98.54%, ranging from 95.79% to 99.67%, and all arrays were included in the analyses. Using CNAG v3.0 software, we performed the genome-wide profiling of CNAs in each tumor primary culture at p1. Globally, the typical "clear cell RCC" genomic signature was confirmed, including deletions on chr 3p and amplifications on chrs 5q and 7 (see Additional File 1), the same signature that we previously described in another set of ccRCC tissue samples [13].

### Genome-wide comparison of DNA profile of primary cultures and original tumor tissues

Our main purpose was to assess if ccRCC primary cultures reflected the genomic profile of tumor tissues from which they derived. First of all, for each tumor primary culture/tissue pair, we calculated the global correlation coefficient (by Spearman regression) between their whole-genome CN profiles. Starting from the HMM-CN state data generated by CNAG, we obtained a mean CN correlation value equal to 0.73 (range 0.30-0.99) (Table 2). This wide range of variation was due essentially to 66SML sample, which presented the lowest CN correlation coefficient (Spearman 0.30). On the other hand, both the concordance index and the Spearman's correlation calculated on CNAG SNP allelic calls gave very high values for all cases (SNP call concordance indexes from 94% to 98% and Spearman's coefficients from 0.87 to 0.96), thus indicating a strong correlation between cultures and corresponding tissues at SNP genotype level, also for the 66SML case (Table 2).

**Table 2:** Estimations of the global correlation between the genomic profile of each primary culture and corresponding parental tissue.

Sample	Spearman's correlation on HMM-CN state	SNP call concordance index	Spearman's correlation on SNP calls
50PC	0.66	98%	0.96
59RG	0.54	98%	0.96
60CC	0.94	94%	0.87
61FG	0.97	98%	0.96
66SML	0.30	96%	0.94
70LS	0.99	98%	0.96
73PG	0.68	97%	0.95
80MLa	0.60	97%	0.93
81BPG	0.90	95%	0.92

**Figure 2:**
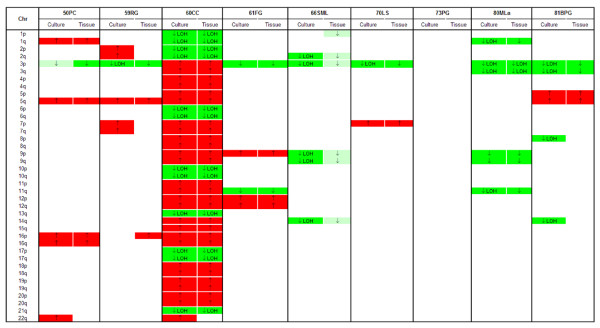
**Copy number alterations and LOH events in ccRCC primary cultures and parental tissues, as calculated by CNAG v3.0 software**. On each chromosomal arm (p, short arm; q, long arm), amplifications (↑) and deletions (↓) and LOH events are reported for all samples. Color labels distinguish CN alterations (CNAs) detected by CNAG and signed in the color-coded "HMM-CN state" track (red for amplifications and dark green for deletions), and CNAs resulting below threshold to be visualized in the HMM-CN track (light green for deletions). Only LOH events reaching significant likelihood to be signed by CNAG in the HMM-LOH track are reported.

Looking at the individual CN profiles produced by CNAG for each sample, 7 out of 9 primary cultures exactly maintained all the DNA alterations presented by the corresponding tumor tissues, or retained the normal CN profile as observed in the original tissue (73PG case) (Figure 2). The remaining two cultures presented the DNA profile of parental tissues except for one CNA: 59RG culture did not show the chr 16p amplification and 66SML culture did not show the chr 1p deletion observed in the corresponding tissues. Moreover, four primary cultures presented additional CNAs on one or two chromosomes, not found in original tissues: 50PC and 60CC cultures had an additional amplification on chr 22q, 59RG showed amplification of chrs 2 and 7, whereas 81BPG showed deletion with LOH on chrs 8p and 14q (Figure 2).

Concerning LOH profile, 60CC culture exactly maintained all the allelic imbalances, with corresponding CN status, found in the original tumor tissue, while 50PC, 61FG and 73PG cultures confirmed the absence of LOH observed in parental tissues (Figure 2). The remaining five primary cultures presented a total of 11 LOH regions that were not detected by CNAG in original tissues because not reaching the LOH likelihood threshold to be classified as statistically significant by the software and thus visualized in the HMM-LOH track. Viceversa, we did not found LOH events occurring in tumor tissues and not confirmed in corresponding cultures.

**Figure 3:**
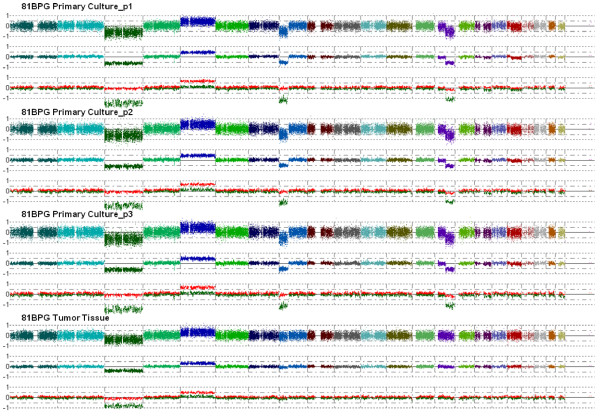
**Whole-genome view of copy number profile in 81BPG primary culture at first (p1), second (p2) and third (p3) confluences, and in corresponding tumor tissue, using CNAG v3.0 software**. Analysis was performed using CNAG v3.0 software, comparing primary culture at each passage and parental tumor tissue to the autologous blood sample. Chromosomes are represented horizontally, from 1 to 22 in different colors, separated by vertical bars. For each sample, the three tracks represent (on log scale): a) "copy number plot": copy number log ratio values of single SNPs; b) "copy number average": copy number log ratio values locally averaged on 10 contiguous SNPs; c) "allele-based analysis": copy number log ratio values for each allele (red and green lines).

Additionally, we performed the whole-genome DNA profiling of 80MLa culture also at second (p2) confluence (see Additional File 2) and of 81BPG culture at second (p2) and third (p3) confluences (Figure 3). In both cases, all the CNAs and LOH observed at p1 were exactly maintained at p2 and p3, and no other alterations occurred along passages. Even the deletions on chrs 8p and 14q found in 81BPG culture at p1, but not in original tissue, were confirmed at p2 and p3 (Figure 3). On the whole, these results indicated that the genomic profile of ccRCC primary cultures highly reflected that of parental tissues and remained stable during the early passages, thus suggesting that these well-characterized primary cultures may be a good *in vitro *model of original tumor tissues.

**Figure 4:**
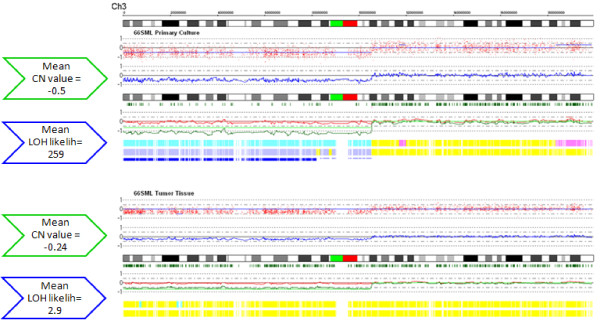
**Visualization of chr 3 in 66SML primary culture (upper panel) and parental tissue (lower panel) using CNAG v3.0 software**. Chromosome 3 is shown from p to q end (from left to right). The upper two graphs represent single SNP copy number data on log2 scale (red dots) and copy number values locally averaged on 10 contiguous SNPs (blue line), whereas copy number values for each allele (red and green lines) are shown below. Green bars in the middle represent heterozygous SNP calls detected by the software comparing each sample to autologous blood. The three bars at the bottom represent the color-coded visualization of HMM-CN state (yellow, diploidy; pink, amplification; light blue, deletion) and of HMM-LOH state (blue, significant LOH; yellow, no LOH), with LOH likelihood indicated by the thickness of the third blue bar. Boxes on the left report mean CN log2ratio values and mean LOH likelihoods calculated for the whole deleted region in primary culture and tissue, respectively.

Moreover, when looking more in detail at the CN profiles calculated by CNAG, we observed that in six primary cultures (59RG, 61FG, 66SML, 70LS, 80MLa, 81BPG) the CN values of aberrant regions were more definite in cultures than in parental tissues and this phenomenon prevalently affected CN loss events. In the 66SML case, this situation was particularly evident. The primary culture presented 4 wide CN loss regions on chrs 2q, 3p, 9 and 14q. As shown in Figure 4 for chr 3p deletion, in primary culture the CNA region was defined by pronounced negative CN values, easily classified by CNAG software as statistically significant CN loss. This deletion was present also in the parental tissue and was visible in the CN profile. However, because of its less pronounced CN values, it did not reach the software threshold to be signed as statistically significant and did not appear in the HMM-CN state track. In Additional File 3 we reported other CN loss regions for 66SML case and two other representative samples: calculating the mean CN values corresponding to each of these deletions, we always observed values more negative in culture than in corresponding parental tissue. This situation reflected also on LOH profiles. In fact, all the 11 LOH events previously mentioned as detected in primary cultures but not in parental tissues occurred in such deleted regions presenting weak CN loss values in tissues. As represented in Figure 4 for chr 3p in 66SML, the software still detected the presence of heterozygous SNP calls in parental tissue (green bars below chromosome ideogram), thus indicating that the hemizygous deletion does not occur in all cells and suggesting the presence of contaminating diploid cells. In all these cases, LOH likelihood consequently decreased below statistical significance threshold and these events were missed to be visualized by CNAG in the HMM-LOH track, as illustrated in Figure 4 (see Additional File 3 for LOH likelihoods of the other deleted regions). Thus, it can be concluded that the increased cell homogeneity of primary cultures, in term of tumor component, in comparison with their parental tumor tissues enabled a better discrimination of CNAs and LOH.

Lastly, we performed the same paired analysis between tumor primary culture and parental tissue using Partek Genomics Suite software, which allows to choose between two alternative CN algorithms. Globally, using the HMM-based algorithm (the same used by CNAG), Partek returned results overlapping with CNAG analysis. Differently, when applying the GS algorithm, Partek was able to retrieve all those CN losses missed to be classified as statistically significant in tissue samples by the CNAG HMM algorithm. This is illustrated for 66SML case in Additional File 4. These results confirmed that in tissue samples these deletions were really present even if less visible than in cultures due to sample heterogeneity, and they did not arise *de novo *in primary cultures.

## Discussion

In this study, we investigated whether the *in vitro *model of ccRCC primary cell cultures, established from surgical tumor specimens, well reproduces the DNA profile of parental tumor tissues, thus allowing a more confident CNAs and LOH discrimination with respect to the original tissues. Tumor cells from ccRCC surgical specimens may be adapted to *in vitro *growth with high efficiency, independently from any clinico-pathological characteristic of patients, as we previously confirmed also in a wide series of well-characterized samples [18,19]. The growth and survival rate of our ccRCC cultures were in agreement with those reported by other authors [14] and proved that our primary cultures grow for the first four passages without difficulties [18]. The cellular composition of our cultures was very homogeneous: more than 90% of cultured cells were of proximal tubular origin, with morphological characteristics typical of ccRCC, and more than 60% of cells were positive for CA9, a biomarker present in almost all ccRCC cases and expressed in most, although not all, of the malignant clear cells of each single positive case [26,28]. When evaluated by Western Blot, CA9 expression was quite strong in all samples, except for 73PG culture where it was undetectable. Notably, about this latter culture, we found an identical normal genomic profile in primary culture and parental tissue in terms of both CNAs and LOH (with the resolution power of the 50K SNP array platform), although the tumor tissue sample, unlike primary culture, showed a CA9 expression by immunohistochemistry (data not shown). This finding is in agreement with another paper reporting a ccRCC case which expressed CA9 in the surgical tissue sample but not in the corresponding primary culture [15]. Moreover, in our experience with a wide series of samples [19], we observed other few ccRCC cases showing a CA9 positivity at tissue level but not in corresponding primary cultures. Excluding tissue sampling mistakes, this behavior might be due to a transitory expression of CA9 during tissue excision. In fact, it is known that surgical conditions, like tissue ischemia after renal artery clamping and subsequent hypoxia, may up-regulate the expression of downstream targets of HIF-1 (hypoxia inducible factor 1), including CA9, that conversely should not be induced under normoxic conditions like those applied for primary cultures [29]. On the other hand, the outgrowth of normal tubular epithelial cells in 73PG culture is unlikely based on the extensive phenotypic characterization we performed on all our cultures [19].

Up to now, karyotypic characterization of RCC primary cultures has been performed only by classical cytogenetic G-banding [30] or CGH technique [23]. In our knowledge, this is the first study applying the Affymetrix SNP array technology to assess at genome-wide level both CNAs and LOH in ccRCC primary cultures. Globally, our cultures confirmed the typical ccRCC genomic signature [13,23,30]. These cultures typically showed alterations on at most 4 or 5 chromosomes. Only two samples made exception: 73PG culture did not show alterations in both CN and LOH profiles, while 60CC showed CNAs on all chromosomes. In particular, 60CC primary culture, as well as its corresponding tissue, showed an atypical ccRCC genomic profile more similar to that of chromophobe subtype (characterized by wide losses on chrs 1, 2, 6, 10, 13, 17 and 21), notwithstanding its typical "clear cell" histology.

It should also be noted that while 73PG specimen derived from a small in size tumor, suggestive of an early stage of neoplastic progression, 60CC specimen derived from the largest one, probably associated with a more advance stage of tumor progression characterized by an aberrant and "clear cell" atypical genomic profile [2].

Up today, very few genomic comparisons between short-term primary cultures and parental tumor tissues have been performed. In glioblastoma, for example, the genomic profile of primary cultures, assessed by array-CGH, resulted considerably different from that of parental tumors, with changes progressively occurring already after 2 weeks of culture, resulting in an inconsistent representation of tumor biology [22]. In melanoma [20] and neuroblastoma [21], instead, using SNP array technology, primary cultures showed to encompass the spectrum of significant alterations present in primary tumors, thus providing a genetically appropriate *in vitro *model for functional genomics characterizations.

Concerning RCC, up till now the comparisons between primary cultures and tumor tissues mainly regarded phenotypic characterizations and proteomic profiling [15,18,31]. A comparison of the genomic profile between primary cultures and parental tissues has been reported only by Sanjmyatav et al. by using traditional CGH [23]. Their DNA profiling showed a poor overlap of CNAs between ccRCC primary cultures and parental tumor tissues. In fact, only 3 out of 8 ccRCC cultures exactly showed the same DNA alterations present in the corresponding tissues. In addition, other 3 cultures did not show any of the CNAs found in the corresponding tissue samples and resulted diploid. Probably, the poor overlap of CNAs obtained by Sanjmyatav et al. [23] was due both to the low sensitivity and resolution level of the technique used (i.e. CGH on metaphase chromosome spreads) and to the not extensive cytological characterization of primary cultures. These findings highlight the importance of a careful phenotypic characterization of primary cultures for a correct interpretation of genomic results.

Globally, these few genomic comparison studies between primary cultures and parental tissues point out that the use of tumor primary cultures as *in vitro *model for genetic analysis or functional studies must be distinctively evaluated tumor by tumor and that in any case it depends on the level of phenotypic characterization of primary cultures.

Looking at the genome-wide CN profiles, our results indicated that 7 out of 9 well-characterized primary cultures exactly reproduced the DNA profile of the corresponding tumor tissues. The other two cultures (59RG and 66SML) maintained all but one the CNAs showed by the original tissues. These findings are in agreement with Lin *et al. *who evidenced that in melanoma some significant alterations present in original tissues (as a deletion on chr 13q) were almost undetectable in cultured cells, despite the landscape of genomic alterations was strikingly similar [20]. However, it must be pointed out that even if primary cultures might have lost some genomic alterations present in original tumor tissues, they probably correspond to some "passenger mutations", that do not confer selective growth advantage to tumor cells and thus might be lost in cultured cells, and not to "driver mutations" [32].

Although the good overlap of CN profiles between each our primary culture and corresponding tissue, we obtained a CN correlation mean value equal to 0.73 (by Spearman regression method), a value just a little higher than that reported for glioblastoma cultures and tissues (Pearson mean 0.62) [22]. The reason is in the wide range of variance we obtained among our 9 cases (range 0.30-0.99), mainly due to the contribution of one case (66SML) on the mean. Although this variability seems too high for conclusive messages, it must be noted that in our series it essentially depends on a software issue. In fact, we used the CNAG "HMM-CN state" data, that is the SNP copy number status inferred by HMM algorithm and visualized in the HMM-CN state track. However, in the presence of a high sample heterogeneity, as in the case of 66SML tissue (illustrated in Figure 4), the HMM-CN state data missed to detect CN aberrations, even if present, because their CN values did not reach the software threshold for a statistical significance. For this reason, the 66SML sample had the lowest CN correlation coefficient between culture and tissue (Spearman 0.30), a value that does not really reflect a poor overlap but only a software constraint.

Similarly to those found in neuroblastoma [21], the SNP call concordance indexes and the Spearman's correlation coefficients calculated on CNAG SNP allelic calls indicated a strong correlation at SNP genotype level between our cultures and parental tissues, 66SML sample included, thus confirming that each primary culture really derived from its corresponding tissue.

Notably, in six samples, CNAs were more evident and better discriminated in primary cultures than in corresponding tumor tissues. This phenomenon principally occurred in deleted regions and it is due to the different cellular composition of the two samples and it was also observed in neuroblastoma tissues and derived cell lines [21]. Tumor tissues are heterogeneous and comprise a mixture of tumor and normal cells (endothelial cells, leukocytes, fibroblasts). For this reason, the copy number values corresponding to DNA alterations of tumor cells are inevitably "diluted" by the diploid values coming from normal cells. Differently, primary cultures were more homogeneous in terms of tumor component, as shown by their phenotypic characterization, and thus presented better defined CNAs. Tumor tissue heterogeneity reflects also on LOH detection. In fact, the software used in the analysis not only detected the allele retained by the tumor, but also the second allele still present in normal cells; in these cases the LOH call will be missed [33]. In our ccRCC primary cultures, instead, hemizygous deletions were accompanied by LOH calls, confirming the great sample homogeneity that enables to discriminate CNAs and LOH. Thus, the presence of LOH calls in deleted regions could be adopted as a parameter to evaluate the degree of tumor sample purity and the level of normal cell contamination and consequently the origin of cultured cells, highlighting the power of SNP array technology with respect to CGH technique. Such an evidence appears more important taking into account that since today there is not a single universally applied method able to certainly make these evaluations [34].

The high cell homogeneity observed in our ccRCC cultures might explain also the additional CNAs found in 4 of them but not in original tissues. The acquisition of these alterations *de novo *during *in vitro *growth is unlikely since cultures were analyzed at first confluence. Moreover, the genomic analysis of some cultures at second and third confluences provided evidence that these cultures did not accumulate further alterations during passages and remained stable for at least 3 weeks. We could therefore conclude that these alterations might fail to be seen in the heterogeneous tumor tissues because present in a very small number of cells (< 20%), thus resulting undetectable for CN analysis algorithms [11,24].

Actually, the true picture of genomic alterations occurring in a tumor can be obtained only performing a genomic analysis directly on tumor cells isolated, for example, by laser capture microdissection, which allows a > 90% of purity [35] and circumvents the problem of DNA alteration "dilution" due to tissue heterogeneity. However, such a useful technical approach has the not negligible inconvenience of providing cellular material not exploitable for eventual subsequent functional studies.

## Conclusions

By performing the genome-wide copy number profiling of a collection of ccRCC primary cultures and corresponding tumor tissues, we demonstrated that these well-characterized primary cultures maintained the genomic alterations of parental tumors. Moreover, their DNA profile remained stable for at least 3 weeks, till to third confluence. Importantly, RCC primary cultures provided greater cell homogeneity and enrichment in tumor component than parental tissues, as proved also by phenotypic characterization, thus enabling a better discrimination of DNA alterations. In this context, SNP array technology demonstrated to be a powerful tool able to confirm the origin of cultured cells and to evaluate sample homogeneity and normal cell contamination. The observation that ccRCC primary cultures retain not only the phenotypic features and the proteomic profile of original tumor tissues but also their genomic profile proves that these short-term cultures are a reliable *in vitro *model that well represents ccRCC genetics and biology and that prospectively could be used for functional approaches.

## Competing interests

The authors declare that they have no competing interests.

## Authors' contributions

IC carried out microarray experiments and bioinformatic analyses. EM and FF performed statistical and bioinformatic analyses. CB, SB, VDS and MAZ prepared primary cultures and performed phenotypic characterizations. EF and SF carried out histological classification of clinical cases and prepared DNA from tissue samples. SS prepared DNA from blood samples.

FM participated in the study design. CB and RAP coordinated and supervised the study and, together with IC and CB, wrote the manuscript. All authors read and approved the final manuscript.

## Pre-publication history

The pre-publication history for this paper can be accessed here:

http://www.biomedcentral.com/1471-2407/11/244/prepub

## Supplementary Material

Additional file 1 **Whole-genome maps of CN alterations and LOH events in the nine ccRCC primary cultures, using CNAG v3.0 software**. Chromosomes are represented from p to q end (from left to right), with cytobands (black and white blocks), centromeres (green blocks) and heterocromatic regions (red and blue blocks). (a) Starting from the HMM-CN state data, regions of CN gain (upper red traces) and CN loss (lower green traces) are represented along each chromosome (from1 to 22). (b) Regions of statistically significant LOH (with LOH likelihood higher than 30) are represented with red traces along each chromosome. Chromosome X was excluded from analyses. Click here for file

Additional file 2 **Whole-genome view of copy number profile in 80MLa primary culture at first (p1) and second (p2) confluences, and in corresponding tumor tissue, using CNAG v3.0 software**. Analysis was performed using CNAG v3.0 software, comparing primary culture at each passage and parental tumor tissue to the autologous blood sample. Chromosomes are represented horizontally, from 1 to 22 in different colors, separated by vertical bars. For each sample, the three tracks represent (on log scale): a) "copy number plot": copy number log ratio values of single SNPs; b) "copy number average": copy number log ratio values locally averaged on 10 contiguous SNPs; c) "allele-based analysis": copy number log ratio values for each allele (red and green lines). Click here for file

Additional file 3 **Copy number and LOH likelihood values for selected deleted regions in primary cultures and corresponding tissues, as calculated by CNAG v3.0 software**. Starting from the Hidden Markov Model (HMM) copy number log2ratios exported for each SNP by CNAG v3.0 software, we calculated the mean CN log ratio value for each region (start and end positions are reported), both in primary cultures and parental tumor tissues. Also, mean LOH likelihood values were calculated for primary cultures and corresponding tissues. In the "CNAG detection" column, we define "CN loss signed" those deletions reaching software threshold to be visualized in the color-coded HMM-CN state track; similarly, "LOH signed" are those events considered as statistically significant by CNAG (with LOH likelihood higher than 30) and thus visualized in the color-coded HMM-LOH track. Click here for file

Additional file 4 **Partek Genomics Suite analysis: CN loss detection in 66SML primary culture and parental tissue by applying the two different algorithms HMM (Hidden Markov Model, the same used by CNAG software) and GS (Genomic Segmentation)**. Analysis was performed starting from CEL intensity files produced by Affymetrix GCOS software, and comparing each ccRCC primary culture and parental tissue to its autologous blood sample. Two different algorithms were used: HMM (Hidden Markov Model) and GS (Genomic Segmentation). Here we reported an example of the different output returned by the two algorithms for 66SML case. On the five chromosomes here displayed (chrs 1p, 2q, 3p, 9, 14q), the CN loss regions, even if clearly visible in the log ratio CN track (upper graph, in log2 scale), failed to be signed by the HMM algorithm in the tissue sample (middle track), exactly as observed in CNAG analysis. Differently, the GS algorithm was able to retrieve all these regions in tissue sample, visualizing them as green bars (bottom track). Click here for file
