# Supplementary material for: Renal cell carcinoma primary cultures maintain genomic and phenotypic profile of parental tumor tissues
Source: BMC Cancer. 2011 Jun 13;11:244. doi: 10.1186/1471-2407-11-244 (PMC3141767; doi:10.1186/1471-2407-11-244)
Supplement: Additional file 1 — Whole-genome maps of CN alterations and LOH events in the nine ccRCC primary cultures, using CNAG v3.0 software. Chromosomes are represented from p to q end (from left to right), with cytobands (black and white blocks), centromeres (green blocks) and heterocromatic regions (red and blue blocks). (a) Starting from the HMM-CN state data, regions of CN gain (upper red traces) and CN loss (lower green traces) are represented along each chromosome (from1 to 22). (b) Regions of statistically significant LOH (with LOH likelihood higher than 30) are represented with red traces along each chromosome. Chromosome X was excluded from analyses. [file 1471-2407-11-244-S1.PDF]

**Additional File 1.** Whole-genome maps of CN alterations and LOH events in the nine ccRCC primary cultures, using CNAG v3.0 software.

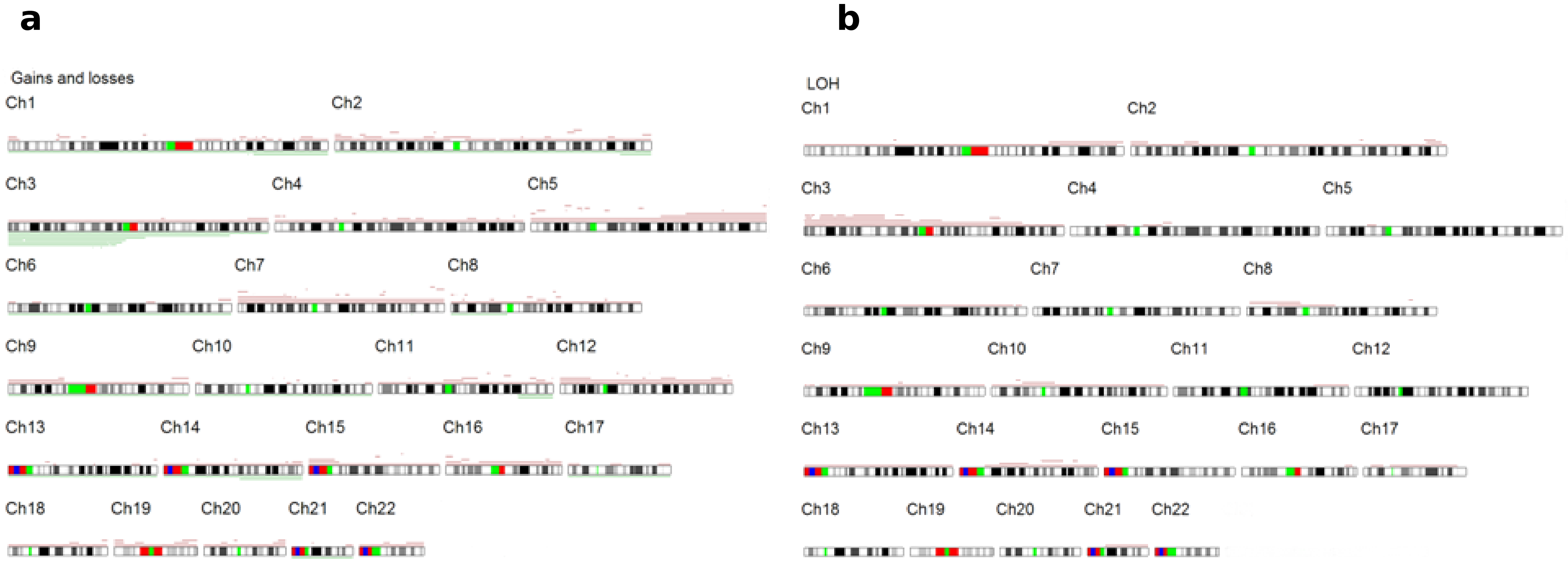


Chromosomes are represented from p to q end (from left to right), with cytobands (black and white blocks), centromeres (green blocks) and heterocromatic regions (red and blue blocks). (**a**) Starting from the HMM-CN state data, regions of CN gain (upper red traces) and CN loss (lower green traces) are represented along each chromosome (from 1 to 22). (**b**) Regions of statistically significant LOH (with LOH likelihood higher than 30) are represented with red traces along each chromosome. Chromosome X was excluded from analyses.
