# Supplementary material for: Renal cell carcinoma primary cultures maintain genomic and phenotypic profile of parental tumor tissues
Source: BMC Cancer. 2011 Jun 13;11:244. doi: 10.1186/1471-2407-11-244 (PMC3141767; doi:10.1186/1471-2407-11-244)
Supplement: Additional file 2 — Whole-genome view of copy number profile in 80MLa primary culture at first (p1) and second (p2) confluences, and in corresponding tumor tissue, using CNAG v3.0 software. Analysis was performed using CNAG v3.0 software, comparing primary culture at each passage and parental tumor tissue to the autologous blood sample. Chromosomes are represented horizontally, from 1 to 22 in different colors, separated by vertical bars. For each sample, the three tracks represent (on log scale): a) "copy number plot": copy number log ratio values of single SNPs; b) "copy number average": copy number log ratio values locally averaged on 10 contiguous SNPs; c) "allele-based analysis": copy number log ratio values for each allele (red and green lines). [file 1471-2407-11-244-S2.PDF]

Additional File 2. Whole-genome view of copy number profile in 80MLa primary culture at first (p1) and second (p2) confluences, and in corresponding tumor tissue, using CNAG v3.0 software.

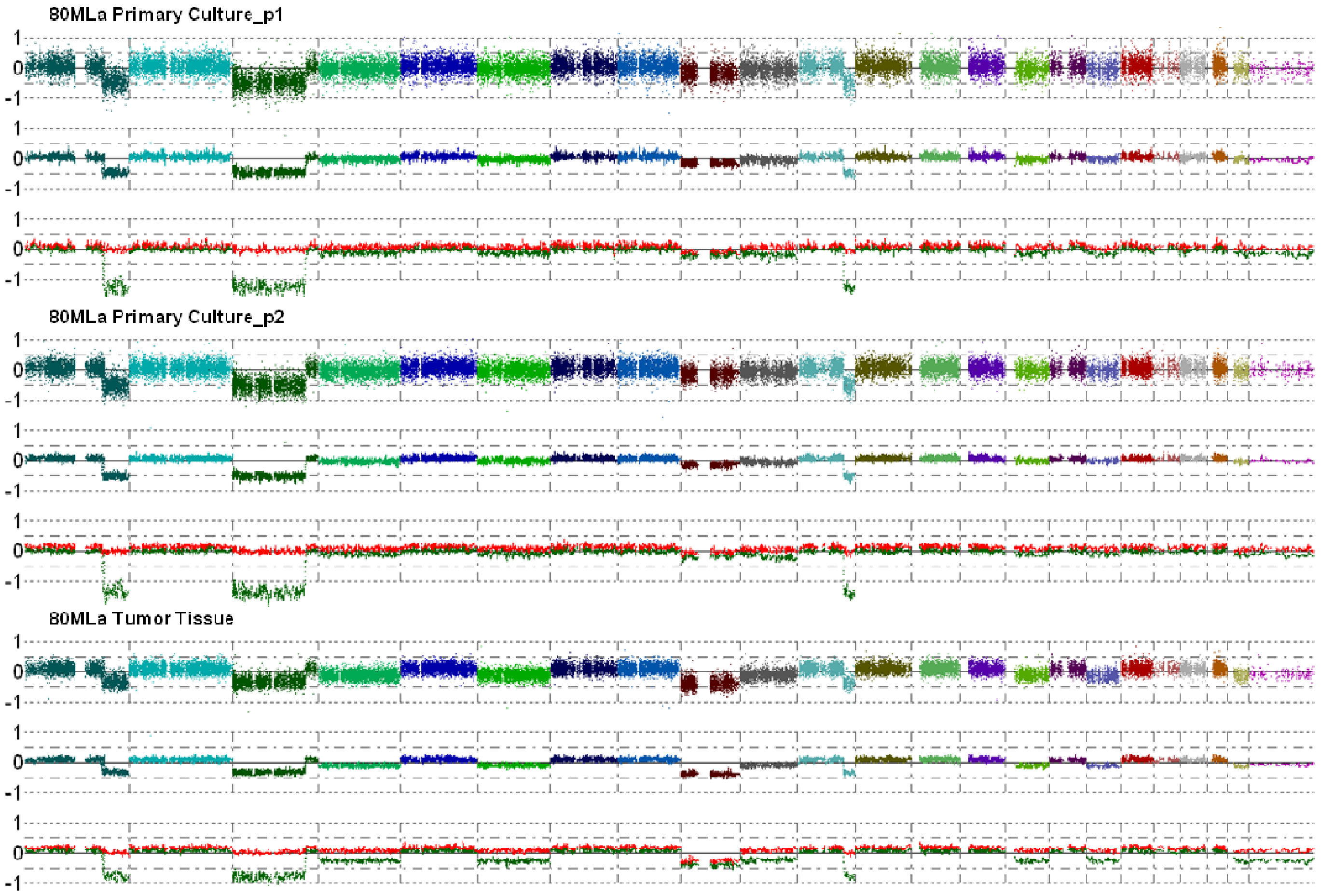

Analysis was performed using CNAG v3.0 software, comparing primary culture at each passage and matched original tumor tissue to the autologous blood sample, as described in Methods section.

Chromosomes are represented horizontally, from 1 to 22 in different colours, separated by vertical bars. For each sample, the three tracks represent (on log scale): a) “copy number plot”: copy number log ratio values of single SNPs; b) “copy number average”: copy number log ratio values locally averaged on 10 contiguous SNPs; c) “allele-based analysis”: copy number log ratio values for each allele (red and green lines).
