# Supplementary material for: Renal cell carcinoma primary cultures maintain genomic and phenotypic profile of parental tumor tissues
Source: BMC Cancer. 2011 Jun 13;11:244. doi: 10.1186/1471-2407-11-244 (PMC3141767; doi:10.1186/1471-2407-11-244)
Supplement: Additional file 3 — Copy number and LOH likelihood values for selected deleted regions in primary cultures and corresponding tissues, as calculated by CNAG v3.0 software. Starting from the Hidden Markov Model (HMM) copy number log2ratios exported for each SNP by CNAG v3.0 software, we calculated the mean CN log ratio value for each region (start and end positions are reported), both in primary cultures and parental tumor tissues. Also, mean LOH likelihood values were calculated for primary cultures and corresponding tissues. In the "CNAG detection" column, we define "CN loss signed" those deletions reaching software threshold to be visualized in the color-coded HMM-CN state track; similarly, "LOH signed" are those events considered as statistically significant by CNAG (with LOH likelihood higher than 30) and thus visualized in the color-coded HMM-LOH track. [file 1471-2407-11-244-S3.PDF]

Additional File 3. Copy number and LOH likelihood values for selected deleted regions in primary cultures and corresponding tissues, as calculated by CNAG v3.0 software.

Sample	Chr	Start Position	End Position	Mean CN LogRatio in culture	Mean CN LogRatio in tissue	Mean LOH likelihood in culture	Mean LOH likelihood in tissue	CNAG detection
61FG	3p	48603	87102814	-0.44	-0.30	6.9	4.5	CN loss signed in culture and tissue, without LOH
	11q	106916419	134173875	-0.43	-0.33	4.9	3.4	CN loss signed in culture and tissue, without LOH
66SML	2q	218401435	242142395	-0.48	-0.22	23	2.2	CN loss (without LOH) signed in culture but not in tissue
	3p	48603	105515576	-0.50	-0.24	259	2.9	CN loss+LOH signed in culture but not in tissue
	9	305185	137012035	-0.50	-0.25	287	3.1	CN loss+LOH signed in culture but not in tissue
	14q	19387587	106241517	-0.50	-0.24	401	4.1	CN loss+LOH signed in culture but not in tissue
70LS	3p	48603	89898956	-0.50	-0.38	33.4	5.1	CN loss signed in culture and tissue, LOH signed only in culture

Starting from the Hidden Markov Model (HMM) copy number log2ratios exported for each SNP by CNAG v3.0 software, we calculated the mean CN log ratio value for each region (start and end positions are reported), both in primary cultures and parental tumor tissues. Also, mean LOH likelihood values were calculated for primary cultures and corresponding tissues. In the "CNAG detection" column, we define "CN loss signed" those deletions reaching software threshold to be visualized in the color-coded HMM-CN state track; similarly, "LOH signed" are those events considered as statistically significant by CNAG (with LOH likelihood higher than 30) and thus visualized in the color-coded HMM-LOH track.
Abbreviations: chr, chromosome; p, short arm; q, long arm; CN, copy number; LOH, loss of heterozygosity.
