# Supplementary material for: Renal cell carcinoma primary cultures maintain genomic and phenotypic profile of parental tumor tissues
Source: BMC Cancer. 2011 Jun 13;11:244. doi: 10.1186/1471-2407-11-244 (PMC3141767; doi:10.1186/1471-2407-11-244)
Supplement: Additional file 4 — Partek Genomics Suite analysis: CN loss detection in 66SML primary culture and parental tissue by applying the two different algorithms HMM (Hidden Markov Model, the same used by CNAG software) and GS (Genomic Segmentation). Analysis was performed starting from CEL intensity files produced by Affymetrix GCOS software, and comparing each ccRCC primary culture and parental tissue to its autologous blood sample. Two different algorithms were used: HMM (Hidden Markov Model) and GS (Genomic Segmentation). Here we reported an example of the different output returned by the two algorithms for 66SML case. On the five chromosomes here displayed (chrs 1p, 2q, 3p, 9, 14q), the CN loss regions, even if clearly visible in the log ratio CN track (upper graph, in log2 scale), failed to be signed by the HMM algorithm in the tissue sample (middle track), exactly as observed in CNAG analysis. Differently, the GS algorithm was able to retrieve all these regions in tissue sample, visualizing them as green bars (bottom track). [file 1471-2407-11-244-S4.PDF]

Additional File 4. Partek Genomics Suite analysis: CN loss detection in 66SML primary culture and parental tissue by applying the two different algorithms HMM (Hidden Markov Model, the same used by CNAG software) and GS (Genomic Segmentation).

Analysis was performed starting from CEL intensity files produced by Affymetrix GCOS software, and comparing each primary culture and matched tumor tissue to its autologous blood sample.

Two different algorithms were used, with the following parameters :

- HMM (Hidden Markov Model) Region Detection: 5 states; at least 3 genomic markers; max probability = 0.98; genomic decay = 0; sigma = 1;
- GS: min genomic markers = 10 SNPs; signal to noise = 0.5; p-value = 0.001.

Here we reported an example of the different output produced by the two algorithms .

On the five chromosomes here displayed (chrs 1p, 2q, 3p, 9, 14q), the CN loss regions, even if clearly visible in the log ratio CN track (upper graph, in log2 scale), failed to be signed by the HMM algorithm in the tumor tissue sample (middle track), exactly as observed in CNAG analysis. Differently, the GS algorithm was able to retrieve all these regions in tissue sample, visualizing them as green bars (bottom track).

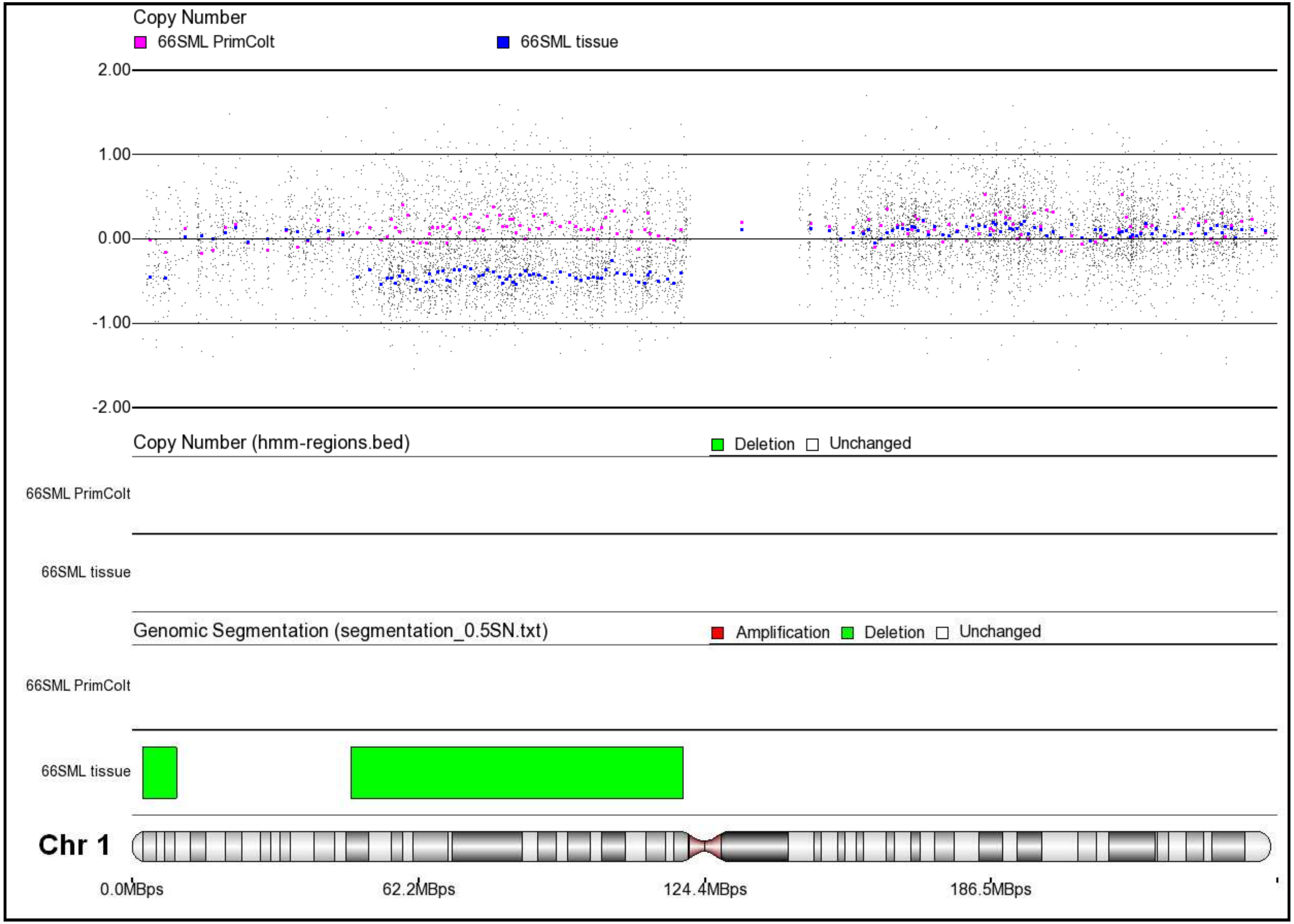
Copy Number
66SML PrimColt
66SML tissue
2.00
1.00
0.00
-1.00
-2.00
Copy Number (hmm-regions.bed)
Deletion
Unchanged
66SML PrimColt
66SML tissue
Genomic Segmentation (segmentation_0.5SN.txt)
Amplification
Deletion
Unchanged
66SML PrimColt
66SML tissue
Chr 1
0.0MBps
62.2MBps
124.4MBps
186.5MBps

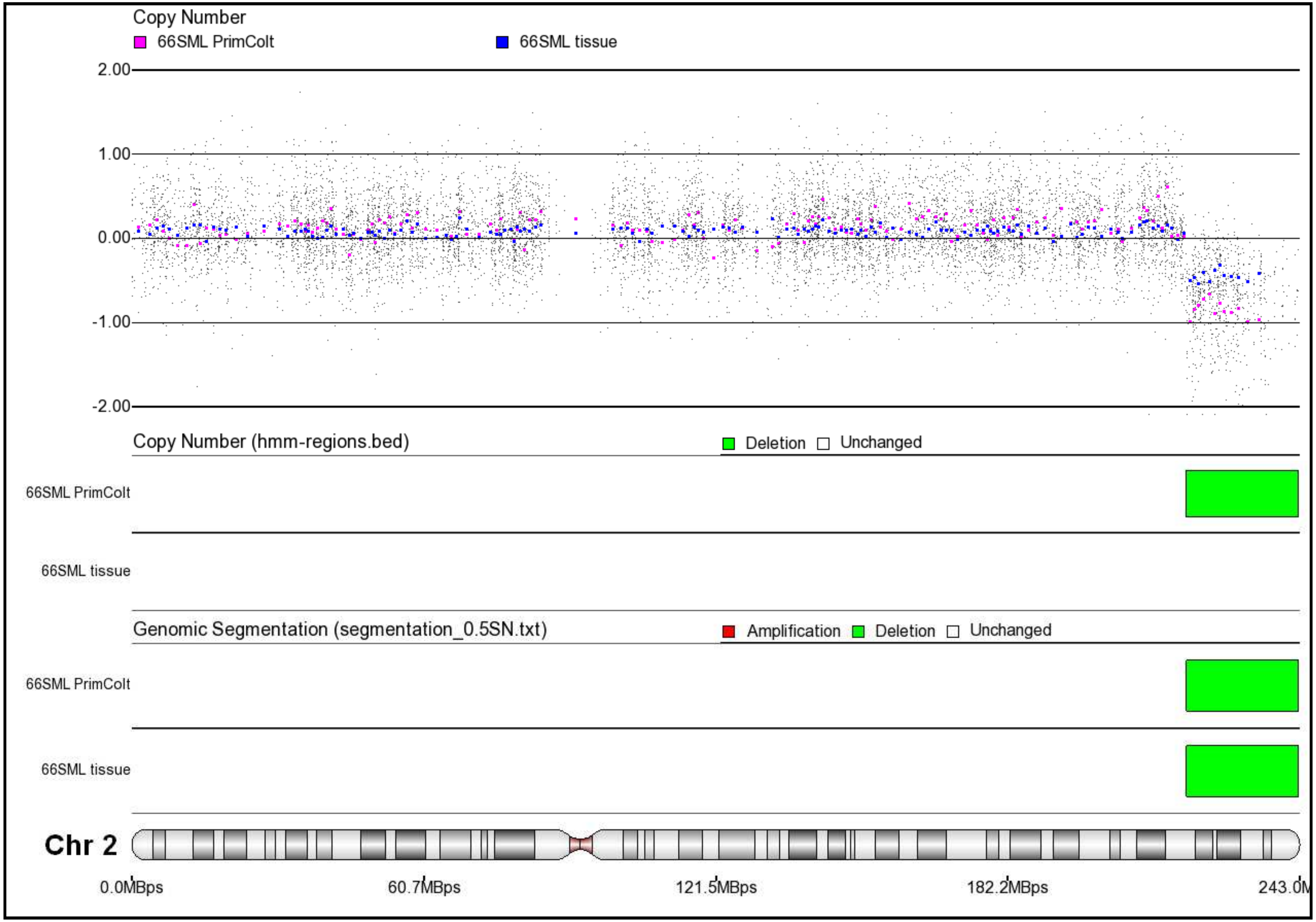

Copy Number
66SML PrimColt
66SML tissue
2.00
1.00
0.00
-1.00
-2.00
Copy Number (hmm-regions.bed)
Deletion
Unchanged
66SML PrimColt
66SML tissue
Genomic Segmentation (segmentation_0.5SN.txt)
Amplification
Deletion
Unchanged
66SML PrimColt
66SML tissue
Chr 2
0.0MBps
60.7MBps
121.5MBps
182.2MBps

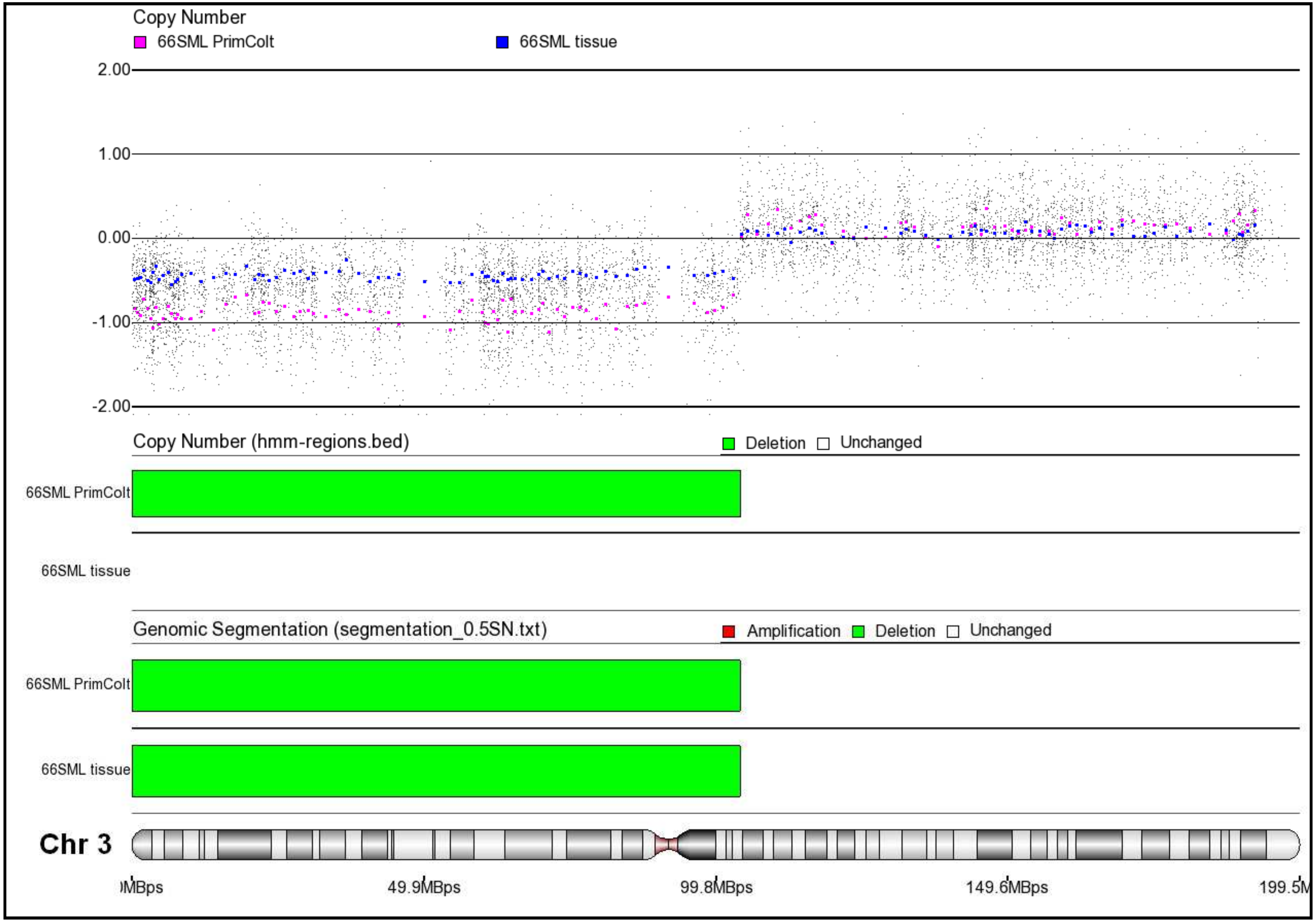
Copy Number
66SML PrimColt
66SML tissue
2.00
1.00
0.00
-1.00
-2.00
Copy Number (hmm-regions.bed)
Deletion
Unchanged
66SML PrimColt
66SML tissue
Genomic Segmentation (segmentation_0.5SN.txt)
Amplification
Deletion
Unchanged
66SML PrimColt
66SML tissue
Chr 3
49.9MBps
99.8MBps
149.6MBps

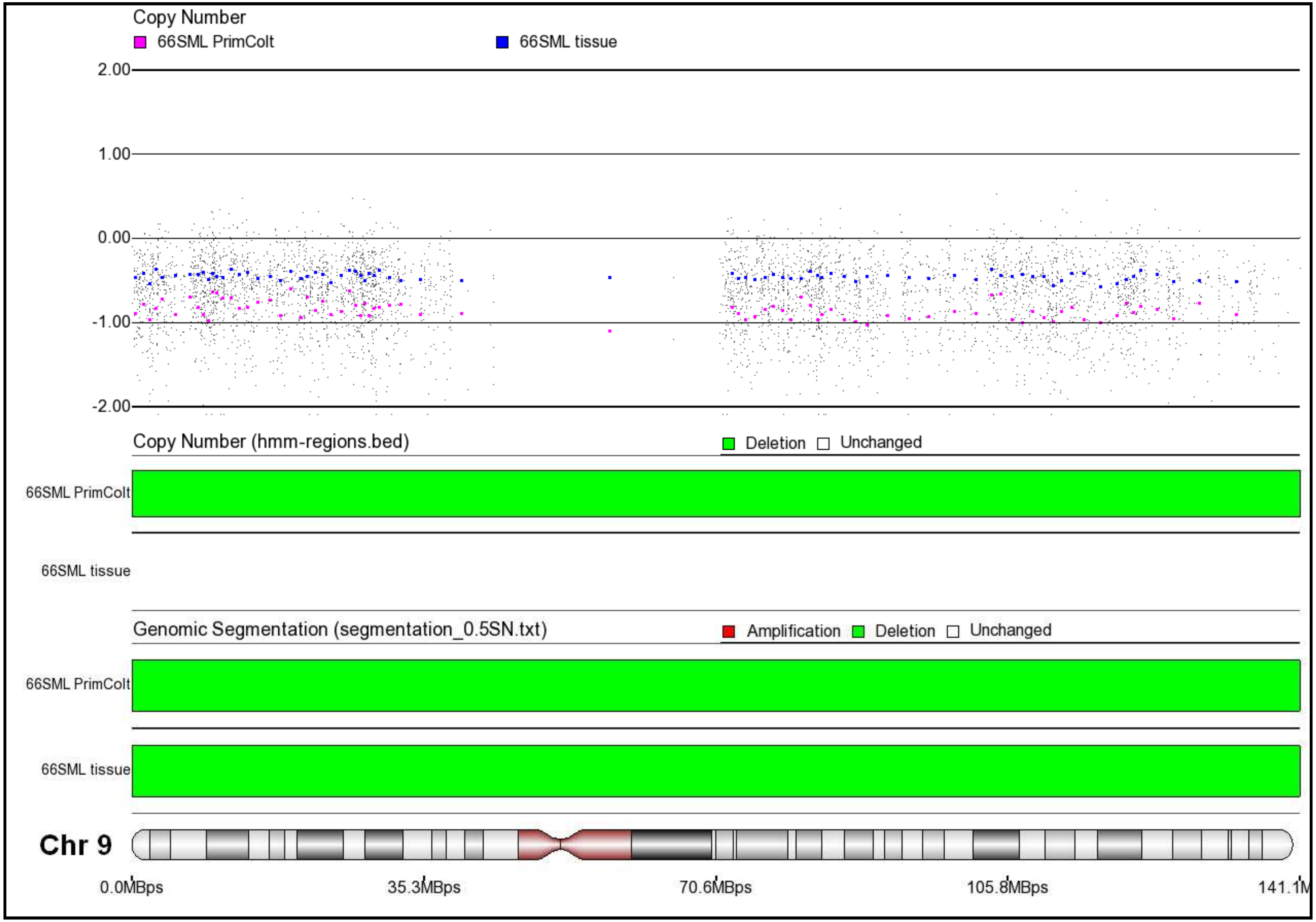

Copy Number
66SML PrimColt
66SML tissue
2.00
1.00
0.00
-1.00
-2.00
Copy Number (hmm-regions.bed)
Deletion
Unchanged
66SML PrimColt
66SML tissue
Genomic Segmentation (segmentation_0.5SN.txt)
Amplification
Deletion
Unchanged
66SML PrimColt
66SML tissue
Chr 9
0.0MBps
35.3MBps
70.6MBps
105.8MBps
141.1M

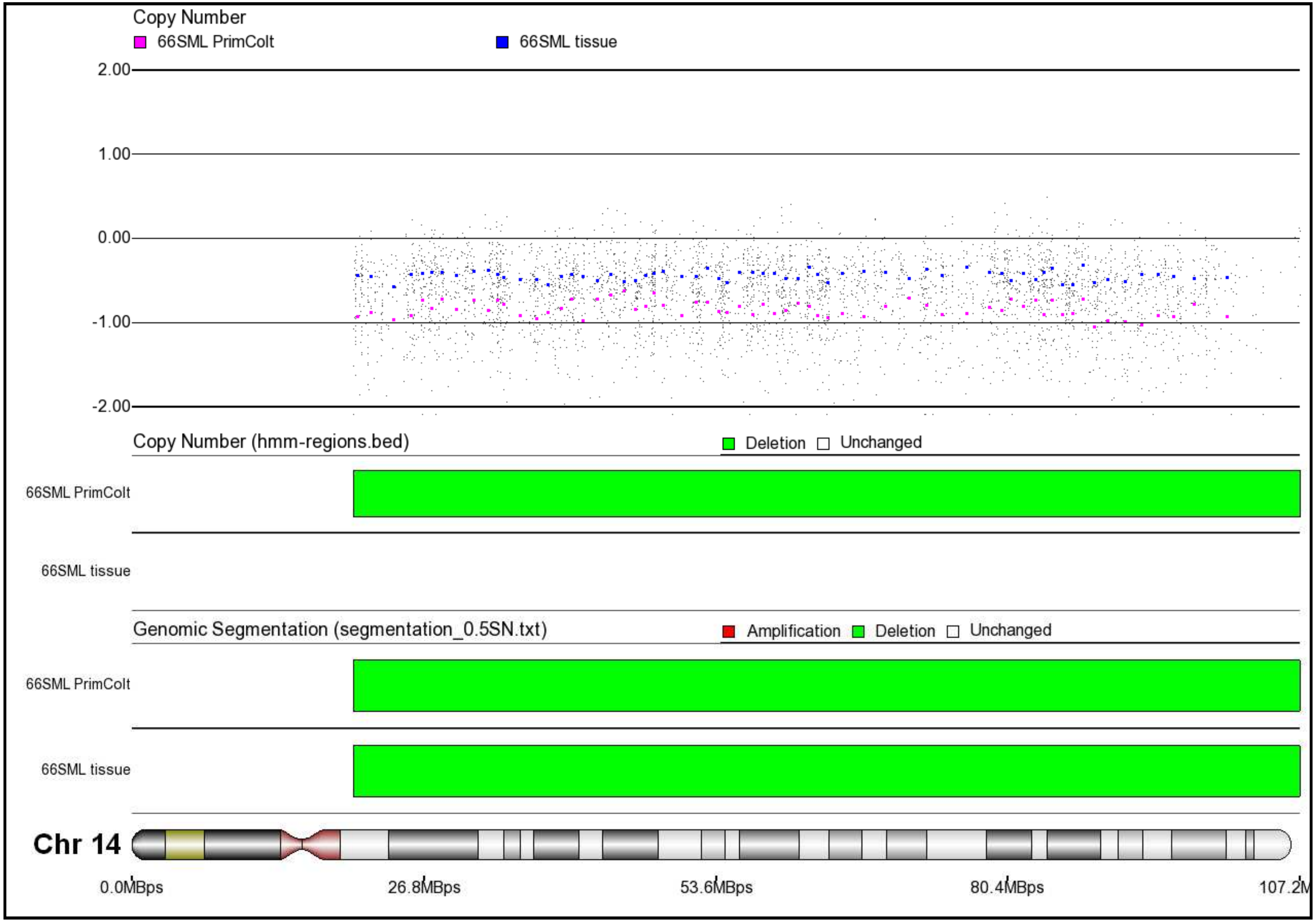
Copy Number
66SML PrimColt
66SML tissue
2.00
1.00
0.00
-1.00
-2.00
Copy Number (hmm-regions.bed)
Deletion
Unchanged
66SML PrimColt
66SML tissue
Genomic Segmentation (segmentation_0.5SN.txt)
Amplification
Deletion
Unchanged
66SML PrimColt
66SML tissue
Chr 14
0.0MBps
26.8MBps
53.6MBps
80.4MBps
107.2M
